# Detecting Bioterror Attacks by Screening Blood Donors: A Best-Case Analysis

**DOI:** 10.3201/eid0908.030079

**Published:** 2003-08

**Authors:** Edward H. Kaplan, Christopher A. Patton, William P. FitzGerald, Lawrence M. Wein

**Affiliations:** * Yale School of Management and Yale Medical School, New Haven, Connecticut, USA; † American Red Cross, Arlington, Virginia, USA; ‡ Stanford University, Stanford, California, USA

**Keywords:** bioterrorism, blood donors, disease outbreaks, probability, stochastic processes, Perspective

## Abstract

To assess whether screening blood donors could provide early warning of a bioterror attack, we combined stochastic models of blood donation and the workings of blood tests with an epidemic model to derive the probability distribution of the time to detect an attack under assumptions favorable to blood donor screening. Comparing the attack detection delay to the incubation times of the most feared bioterror agents shows that even under such optimistic conditions, victims of a bioterror attack would likely exhibit symptoms before the attack was detected through blood donor screening. For example, an attack infecting 100 persons with a noncontagious agent such as *Bacillus anthracis* would only have a 26% chance of being detected within 25 days; yet, at an assumed additional charge of $10 per test, donor screening would cost $139 million per year. Furthermore, even if screening tests were 99.99% specific, 1,390 false-positive results would occur each year. Therefore, screening blood donors for bioterror agents should not be used to detect a bioterror attack.

The health and economic consequences of an extensive bioterror attack could be severe (*1*–*5*); thus, early detection of an otherwise silent bioterror attack is of obvious importance (*6*). Ongoing developments in rapid testing for potential bioterror agents (*7*–*10*) led us to consider whether screening blood donors to detect a bioterror attack with the most feared bioterror agents (*11*) could prove useful. The rationale for screening blood donors rests is twofold. First, blood donors are numerous, and donations are uniformly spread over time and throughout the population. In the United States, approximately 13.9 million blood donations are made each year (*12*); thus, the annual number of donations roughly equals 5% of the 286 million population. Second, in the absence of specific information regarding how such an attack might target the population, we can assume that blood donors are as likely to be infected in a bioterror attack as nondonors. In a sizeable attack, infected donors might donate blood before their infections have been detected medically. Screening donated blood for bioterror agents could therefore serve to detect an attack sooner than would otherwise be possible.

However, the cost of screening donations is proportional to the number of donations tested, in addition to the resources expended investigating false alarms. To investigate these issues, we developed a model for bioterror attack detection under assumptions favorable to donor screening, for if such best-case assumptions fail to justify screening donors, more realistic assumptions will also. In particular, we initially assume that the screening test used is perfectly specific, which removes the possibility of false alarms, and compare the time required to detect an attack through donor screening to the incubation periods for various bioterror agents to see whether donor screening leads to more rapid detection than simply observing symptomatic cases. We then consider tests with imperfect specificity, examine the false-alarm rate that would result from donor screening, and compare this rate to the true-positive rate for blood donations.

## Methods

Though blood tests with the ability to detect agents such as smallpox virus or *Francisella tularensis* within days after infection do not exist at present, research to develop such sensitive tests is under way (*7*–*10*). To analyze whether screening donors might meaningfully shorten the time required to detect an attack were such tests available, we developed a probabilistic model that joins the workings of a screening test, blood donation, and epidemic spread under assumptions that deliberately favor attack detection through donor screening (see Appendix). In the model, the sensitivity of a screening test is determined by a (random) window period *W* with mean *ω* days that must transpire before a person infected at time 0 can be detected as infected. Test sensitivity thus depends on the time from infection until testing. Though the model can accommodate any probability distribution desired, we take *W* to follow an exponential distribution in our examples, an assumption that favors early detection (since the exponential likelihood is maximized at *W*=0, that is, *no* detection delay, and declines as *W* increases). We assume initially that the screening test is perfectly specific, though we will relax this assumption later.

A bioterror attack at time 0 infects *I*(*0*)*=Np* persons in a population of size *N* (where *p* is the fraction of the population initially infected). We assume that everyone in the population has the same probability *p* of infection due to the attack, that is, the attack does not target the population in a manner that would make blood donors more or less likely to be infected than nondonors. Given that the total number of blood donations over time results from the independent actions of individual blood donors, the aggregate number of blood donations over time was modeled as a Poisson process (*13*) with rate λ=*kN*, where *k* is the mean number of blood donations per person per unit of time. If the agent used in the attack is contagious, secondary infections spread according to an epidemic model, governed by a reproductive number *R*_0_ (number of secondary infections per initial index case) and an exponentially distributed duration of infectiousness with mean *r*^-1^. To favor donor screening, we deliberately exclude an explicit latent period (during which an infected person is not infectious). These assumptions imply that infections in the population will grow exponentially with rate (*R*_0_ – 1)*r* postattack (*14*), an assumption that further favors donor screening as the number of blood donors who are infected (and by ignoring latent periods, infectious) will grow exponentially at the same rate, leading to earlier detection via donor screening than would occur otherwise.

We assume that the attack is detected once a single infected donation tests positive for infection with a bioterror agent, another assumption favorable to donor screening, which enables us to derive the probability distribution of the time required to detect a bioterror attack of a given magnitude. However, to demonstrate the extent to which we have “stacked the deck” in favor of blood donor screening, we relax the assumption of perfect test specificity for noncontagious agents. We assume fixed attack rates and disaster response and recovery periods, which together determine the fraction of time during which infected donations can occur. This assumption allows us to model the rate of false alarms per unit of time and compare this to the rate of true-positive alarms.

## Results

**Figure 1:**
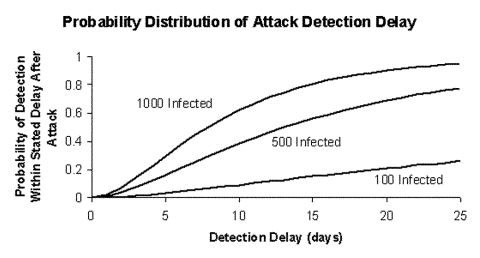
Probability distribution of attack detection delay for a noncontagious agent. Blood donations occur at rate *k*=0.05 per person per year, the screening test has a mean window period of *ω*=3 days, and initial attack sizes range from 100 through 1,000 infections.

**Figure 2:**
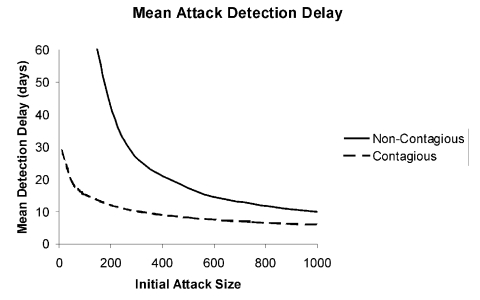
Mean attack detection delays for noncontagious (solid) and contagious (dashed) agents as a function of the initial attack size. Other parameters set as in Figures 1 and 3.

For initial attacks ranging from 100 to 1,000 infections, Figure 1 shows the probability distribution of the attack detection delay for a noncontagious agent that would result from using a blood-screening test able to detect infections an average of *ω* = 3 days after infection (an optimistic assumption, given that such tests do not exist at present), assuming that blood donations arrive at rate *k* = 0.05 per person per year, the average rate for blood donation in the United States (*12*). The results are not encouraging: for an attack that infects 100 persons, the chance of detecting the attack through blood donor screening within 25 days is 26%; even for a large attack that infects 1,000 persons, the median time to detect the attack is 8 days. Figure 2 (solid curve) shows the mean delay in attack detection as a function of the initial attack size for a noncontagious agent. For an initial attack that affects 1,000 persons, the mean time to detection is 10 days, while for an attack that affects 100 persons, the mean time to detection is 76 days. In most infected persons, symptoms would develop during this period, leading to earlier detection of an attack than blood donor screening would allow, even when potential delay from misdiagnosis or failure to recognize symptoms is accounted for (Table 1; compare to incubation times from infection through symptoms for *Bacillus anthracis* and *Clostridium botulinum*, two noncontagious agents). That we have deliberately made assumptions favorable to blood donor screening strengthens this finding, for the actual time required to detect an attack by means of donor screening would be longer than reported above.

**Table 1:** Incubation periods from infection through symptoms for Centers for Disease Control category A agents

Agent	Incubation time (days)
*Bacillus anthracis*	<7
*Clostridium botulinum*	0.5–1.5
*Yersinia pestis*	1–6
Smallpox virus	7–17
*Francisella tularensis*	3–5
Hemorrhagic fever viruses	2–21 (Ebola); 5–10 (Marburg)

Source (*11*).

**Figure 3:**
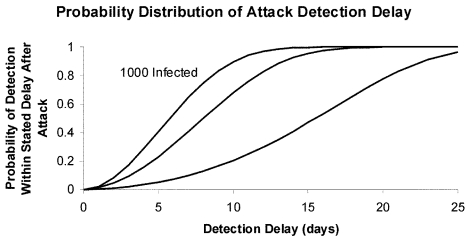
Probability distribution of attack detection delay for a contagious agent. Blood donations occur at rate *k*=0.05 per person per year, the screening test has a mean window period of *ω*=3 days, the reproductive number *R*_0_=3, the mean duration of infectiousness *r*^-1^=14 days, and initial attack sizes range from 100 through 1,000 infections.

If we also assume that *ω*=3 days and *k*=0.05 per person per year, Figure 3 shows the distribution of delays in attack detection that would result from a contagious agent characterized by *R*_0_=3 and *r*^-1^=14 days (parameters suggestive of smallpox [*3**,**11**,**15*] and perhaps Ebola virus [*11*]). Because additional infections are transmitted to susceptible persons, the probability of detecting an attack within any given period is greater than for a noncontagious agent. Consequently, for a given initial attack size, the attack detection delay distribution is shorter for a contagious agent, as is clear from Figure 3. However, symptoms would develop in many infected persons, and such infections would be recognized before blood donor screening would uncover an attack. Under our best-case assumptions, an attack that initially infects 100 persons would still require 15 days on average before donor screening would detect the attack, while an initial attack infecting 1,000 persons would require 6 days until detection on average (Figure 2).

Treating the range of incubation times from infection through symptoms (Table 1) as 99% probability intervals from agent-specific lognormal distributions, in the case of smallpox one would expect to see five symptomatic cases after 7 days, while more than half of those initially infected with Ebola virus would progress to symptoms within 1 week. The incubation times for plague and tularemia are much shorter (Table 1), but even after increasing *r* to compensate for this in our model many of those infected would exhibit symptoms before the bioterror event was detected through tests of the blood supply (results not shown). Again, considering that we have made assumptions that favor donor screening—that the test has an exponentially distributed window period that detects infection after 3 days on average, that donor screening detects the attack after the first donor tests positive, that there is no latent period from infection through infectiousness, and that a postattack epidemic grows exponentially—donor screening as a method of attack detection does not seem competitive with simple observation of symptomatic case-patients.

Until now, we have assumed that screening occurs with perfect specificity, which eliminates false-positive results as a consequence. However, if false-positive test results can occur, they will occur frequently. Table 2 reports the false-alarm rates that would occur for tests of different specificities for a noncontagious agent, if one assumes that all 13.9 million annual blood donations are tested, that on average one bioterror attack takes place per year (a rate all would agree is unrealistically high), that on average 1 month is required to respond to and recover from an attack (so infected donations can occur for up to 1 month after an attack), and that each attack infects 1,000 persons. Even with 99.99% specificity, an average of 1,390 false-positive results would occur per year; at 99% specificity, the average would be 139,000 false-positive results per year.

**Table 2:** False-alarm rates with test specificities as shown^a^

Specificity (*s*)	Annual false-alarm rate (FAR)
0.9	1,390,000
0.99	139,000
0.999	13,900
0.9999	1,390

^a^If one assumes 13.9 million annual blood donations tested, an average of one bioterror attack per year that infects 1,000 persons with a noncontagious agent, and a 1-month response and recovery period during which infected donations continue to arrive.

In addition to the resources wasted in investigating so many false alarms, a “crying wolf” mindset could diminish the attention paid to all screening test results, increasing the chance of missing a true-positive test result. That this latter possibility could well occur seems clear because with the attack rate and duration of response and recovery assumed above, one would expect only 3.7 donations with true-positive results each year (again, presuming an exponentially distributed window period with mean *ω=* 3 days). Also, though lowering the attack rate below one per year to more realistic levels would have no effect on the false-positive rate, the number of donations with true-positive results would fall. Similarly, reducing the duration of the postattack response and recovery during which infected donations can still occur would have essentially no impact on the false-positive rate, while again lowering the number of donations with true-positive results.

## Conclusion

We have argued that even under assumptions deliberately favorable to blood donor screening, an attack was unlikely to be detected earlier through donor screening than from observing symptomatic case-patients. We have also shown that imperfect test specificity could overwhelm the blood collection system with false-positive results. In addition, the costs of screening apply to all blood donations tested: even if the cost of screening were as low as an incremental $10 per test, screening all blood donations in the United States to detect a bioterror attack would cost an additional $139 million per year at current donation rates. Total costs would be even higher when the resources that would be expended investigating false-positive results are considered. For all of these reasons, blood donors should not be screened for bioterror agents for the purpose of detecting a bioterror attack.

E.H.K. was supported in part by Yale University’s Center for Interdisciplinary Research on AIDS via Grant MH/DA56826 from the U.S. National Institutes of Mental Health and Drug Abuse.

## Supplementary Material

Appendix Detecting Bioterror Attacks by Screening Blood Donors: A Best-Case Analysis
