# Supplementary material for: Detecting Bioterror Attacks by Screening Blood Donors: A Best-Case Analysis
Source: Emerg Infect Dis. 2003 Aug;9(8):909–14. doi: 10.3201/eid0908.030079 (PMC3020608; doi:10.3201/eid0908.030079)
Supplement: Appendix — Detecting Bioterror Attacks by Screening Blood Donors: A Best-Case Analysis [file 03-0079-app-s1.pdf]

Appendix

We consider a single bioterror attack that infects a proportion *p* of the population at time 0. To model test sensitivity, we presume that a blood test administered to a person *t* days after becoming infected will test positive for infection with probability $F_W(t)$, where *W* refers to the window period of the test. In our examples we assume that *W* follows the exponential distribution with mean *?* days, that is, $F_W(t) = 1 - e^{-t/?}$, though the model allows assessment for any window period distribution. We set *?*=3 days in our examples.

The probability that a randomly selected member of the population would test positive *t* days after the attack is then given by

$$\pi(t) = pF_W(t) + \int_0^t \iota(u) F_W(t-u)\,du \qquad (1)$$

where $\iota(u)$, the per-capita rate of infection due to transmission after attack (but before detection) grows exponentially as

$$\iota(u) = pR_0 r \exp((R_0 - 1)ru) \qquad (2)$$

as explained following equation 8 below. In equation (2), R_0 is the reproductive number specifying the number of secondary infections transmitted by an initially infected individual early in the outbreak, while r^{-1} is the mean duration of infectiousness (14). We set R_0=3 and r^{-1}=14 days in our contagious examples, parameters suggestive of smallpox (3,15), while results for attacks with noncontagious agents are obtained by setting R_0=0. Note that p(*t*) is proportional to *p*, the fraction of the population initially infected in the attack.

Due to the superposition of many individually arriving donors (13), we assume that in the aggregate, blood donations occur in accord with a Poisson process with rate l per unit of time. We set *l* =*kN* for some constant *k*, that is, the blood donation rate is proportional to the size of the population (*k*=0.05 to represent the average U.S. donation rate [12] in our examples). We further assume that donors are no more or less likely to have been infected than nondonors. The number of blood donations that would test positive within time t of the attack then follows a Poisson distribution with mean

$$\rho(\tau) = \int_0^\tau \lambda \pi(t)\,dt. \qquad (3)$$

Note that since p(*t*) is proportional to *p* while l is proportional to *N*, ?(t) is proportional to *I*(0)=*Np*, the initial attack size. Thus the ability to detect a bioterror attack by means of blood donor screening when blood donation occurs at a rate proportional to the population is directly related to the initial number of persons infected in the attack, independently of the size of the population.

The probability that at least one blood donation would test positive and detect the attack within t days is given by

$$D(\tau) = 1 - \exp(-\rho(\tau)) \qquad (4)$$

while the expected time required to detect such an attack equals

$$E[\text{Attack Detection Delay}] = \int_0^\infty \exp(-\rho(\tau))d\tau$$ (5)

because the expected value of a nonnegative random variable equals the integral of its survivor function, as is well-known. Since ?(t) is proportional to the initial attack size, the probability of detecting an attack within any fixed time interval increases with the initial attack size, while the expected time required to detect an attack decreases with the size of the attack.

In the event of an attack at time 0 with a contagious agent, we approximate the progress of the resulting epidemic with the standard model

$$\frac{dI(t)}{dt} = \beta I(t)[N - I(t)] - rI(t)$$ (6)

where N is the population size, and

$$\beta = \frac{R_0 r}{N}$$ (7)

is the disease transmission rate (14). Persons infected in this model immediately become infectious and remain so for r^{-1} time units on average; thus; no latent period occurs during which a person is infected but not infectious. Early in the epidemic we have

N–$1(t)$˜N, which as usual leads to exponential growth in the number of infections as

$$I(t) = I(0)\exp((\beta N - r)t) = I(0)\exp((R_0 - 1)rt).$$ (8)

Note that the per-capita transmission of infection before the detection of the attack in this model is given by $\beta NI(t)/N = pR_0 r\exp((R_0 - 1)rt)$ as in equation 2.

The sensitivity of the attack detection delay to the parameters of this model can be determined directly from the mathematics above. To summarize, the time to detect an attack via blood donor screening will decrease if, *ceteris paribus*, any of the following parameters increase: the initial number of infections, $I(0)$, the per capita blood donation rate (k), the reproductive number (R_0), and the disease progression rate (r). Increasing the mean window period of the screening test (*?*) would lengthen the time required to detect an attack.

The screening test employed is perfectly specific in the analysis above, which obviates the problem of false alarms by assumption. We now relax the assumption of perfect specificity and instead assume that an uninfected donation will test negative with probability s, where s is the specificity of the test. With this new assumption, uninfected donations will test positive with probability 1-s, which leads to false-positive results.

To compare false-positive and true-positive rates for noncontagious agents, we adopt an alternating renewal process model (13) of bioterror attack and recovery (a similar analysis could be conducted for contagious agents, but little insight can be gained from doing so). Under normal circumstances, we assume that attacks occur at a mean rate of a per unit time. Once an attack occurs, we assume that d time units are required for response and recovery (clearly d would depend upon the time required to detect an attack, which in turn could be influenced by donor screening, but this effect is minor and not essential for the main results reported). Infected donations can only occur during the response and recovery period,

while to simplify the analysis, we presume that no further attacks ensue during the recovery period (indeed, multiple attacks could simply be modeled within this framework as one larger attack). Again for simplicity, we further assume that blood donations occur at the constant rate *l= kN* over time, and that any attack infects a fraction *p* of the population.

With these assumptions, it follows immediately that the fraction of time occupied by response and recovery, which coincides with the fraction of time during which infectious donations can occur, is given by

$$f = \alpha\delta/(1+\alpha\delta). \qquad (9)$$

It follows that the false-alarm rate, FAR (i.e., the mean number of noninfected donations that falsely test positive), is equal to

$$FAR = kN(1-s)[1-f+f(1-p)] = kN(1-s)(1-fp) \qquad (10)$$

for all donations that test positive do so falsely under normal circumstances, while during the response and recovery period, a fraction (1-*p*) of donations will be noninfected, and of these

(1-*s*) will falsely test positive.

To obtain a simple formula for the true-positive donation rate, note first that the overall attack rate per unit time is given by

$$\alpha' = \alpha(1-f) \qquad (11)$$

because, by assumption, attacks do not occur during the response and recovery period. Since ?(d)) infected donations will occur on average during the response and recovery period (where ?(d)) is given by equation [3]), the overall true-positive donation rate (*TPDR)* is given by

$$TPDR = \alpha'\rho(\delta). \qquad (12)$$

In the text, we report results for d=1 month and α' =1 attack per year, but again the sensitivity of the results to the model parameters is clear from the mathematics: reducing either the attack rate or the duration of response and recovery serves to reduce the true-positive donation rate while marginally increasing the false-positive rate; increasing test specificity obviously reduces the false-alarm rate.
